# Impact of Antibiotic Duration on Gut Microbiome Composition and Antimicrobial Resistance: A Substudy of the BALANCE Randomized Controlled Trial

**DOI:** 10.1093/ofid/ofaf137

**Published:** 2025-03-14

**Authors:** Eric Armstrong, Maria Kulikova, Noelle Yee, Asgar Rishu, John Muscedere, Stephanie Sibley, David Maslove, J Gordon Boyd, Gerald Evans, Michael Detsky, John Marshall, Linda R Taggart, Jan O Friedrich, Jennifer L Y Tsang, Erick Duan, Karim Ali Firdous, David McCullagh, Aidan Findlater, Rob Fowler, Nick Daneman, Bryan Coburn

**Affiliations:** Toronto General Hospital Research Institute, University Health Network, Toronto, Ontario, Canada; Toronto General Hospital Research Institute, University Health Network, Toronto, Ontario, Canada; Toronto General Hospital Research Institute, University Health Network, Toronto, Ontario, Canada; Sunnybrook Research Institute, Sunnybrook Health Sciences Centre, Toronto, Ontario, Canada; Department of Critical Care Medicine, Queen's University, Kingston, Ontario, Canada; Department of Critical Care Medicine, Queen's University, Kingston, Ontario, Canada; Department of Critical Care Medicine, Queen's University, Kingston, Ontario, Canada; Department of Medicine, Queen's University, Kingston, Ontario, Canada; Department of Critical Care Medicine, Queen's University, Kingston, Ontario, Canada; Division of Neurology, Department of Medicine, Queen's University, Kingston, Ontario, Canada; Division of Infectious Diseases, Department of Medicine, Queen's University, Kingston, Ontario, Canada; Critical Care Medicine, Mount Sinai Hospital, Unity Health Toronto, Toronto, Ontario, Canada; Critical Care Medicine, Mount Sinai Hospital, Unity Health Toronto, Toronto, Ontario, Canada; Surgery, Unity Health Toronto, Toronto, Ontario, Canada; University of Toronto, Toronto, Ontario, Canada; Division of Infectious Diseases, Department of Medicine, University of Toronto, Toronto, Ontario, Canada; Division of Infectious Diseases, Department of Medicine, Unity Health Toronto, St Michael's Hospital, Toronto, Ontario, Canada; Critical Care, Unity Health Toronto, St Michael's Hospital, University of Toronto, Toronto, Ontario, Canada; Medicine, Unity Health Toronto, St Michael's Hospital, University of Toronto, Toronto, Ontario, Canada; Niagara Health Knowledge Institute, Niagara Health, St. Catharines, Ontario, Canada; Division of Critical Care, Department of Medicine, McMaster University, Hamilton, Ontario, Canada; Division of Infectious Diseases, Niagara Health, St. Catharines, Ontario, Canada; Division of Infectious Diseases, Niagara Health, St. Catharines, Ontario, Canada; Infectious Diseases, McMaster University, Hamilton, Ontario, Canada; Sunnybrook Research Institute, Sunnybrook Health Sciences Centre, Toronto, Ontario, Canada; University of Toronto, Toronto, Ontario, Canada; Sunnybrook Research Institute, Sunnybrook Health Sciences Centre, Toronto, Ontario, Canada; University of Toronto, Toronto, Ontario, Canada; Division of Infectious Diseases, Department of Medicine, University of Toronto, Toronto, Ontario, Canada; Toronto General Hospital Research Institute, University Health Network, Toronto, Ontario, Canada; Division of Infectious Diseases, Department of Medicine, University of Toronto, Toronto, Ontario, Canada

**Keywords:** antibiotics, antimicrobial resistance, antimicrobial stewardship, bacteremia, microbiome

## Abstract

**Background:**

Maintaining a diverse gut microbiome and minimizing antimicrobial resistance gene (ARG) carriage through reduced antibiotic utilization may decrease antimicrobial resistance. We compared gut microbiome disruption and ARG carriage following 7 or 14 days of antibiotics for treatment of bacteremia in a substudy of the BALANCE randomized controlled trial.

**Methods:**

The BALANCE randomized controlled trial enrolled 3631 participants with bacteremia, who were randomized 1:1 to receive 7 or 14 days of antibiotics. Rectal swabs were collected from 131 participants and analyzed with metagenomic sequencing to characterize the gut microbiome and ARGs. The primary outcome was change in gut microbiome diversity at day 7 vs 14.

**Results:**

Forty-one participants (n = 28 in the 14-day group, n = 13 in the 7-day group) had samples available for the primary analysis, with an imbalance in piperacillin-tazobactam exposure between groups. Change in gut microbiome diversity at day 7 vs 14 was comparable between the 14-day group (median, 0.07; IQR, −0.46 to +0.51) and 7-day group (median, 0.19; IQR, −0.77 to +0.22; *P* = .49). Change in ARG abundance at day 7 vs 14 did not differ by treatment duration, nor did the abundance of individual ARGs. We did not observe any change in gut microbiome diversity or ARG carriage at enrollment vs day 7.

**Conclusions:**

In this subset of patients from the BALANCE randomized controlled trial, we did not detect greater gut microbiome disruption or ARG carriage among participants who received 14 vs 7 days of antibiotics, but we were limited by small sample size and imbalances between groups.

Antimicrobial resistance is a threat to health globally and is responsible for significant morbidity, mortality, and health care costs [1]. Antibiotic use exerts selective pressure for antimicrobial-resistant organisms, and unnecessary or excessive use is therefore a critical target to curb the spread of antimicrobial resistance [2, 3]. Antibiotic stewardship seeks to optimize antibiotic treatment to minimize harms and retain efficacy, with the dual goals of improving patient outcomes and reducing the spread of antimicrobial resistance [4, 5]. Although various clinical studies have evaluated the impact of antibiotic duration on the gut microbiome and antimicrobial resistance [6–8], these studies were observational and thus limited in their ability to assess the causal impact of antibiotic duration on gut microbiome disruption.

The gut microbiome is a reservoir for antimicrobial resistance genes (ARGs), collectively referred to as the gut “resistome” [9]. Acquisition of ARGs by pathogens can occur by mutation or horizontal gene transfer [10]. Maintenance of a healthy diverse gut microbiome can resist expansion of pathogens and their acquisition of ARGs. Conversely, antibiotic treatment can disrupt the gut microbiome and exert selective pressure for ARGs, simultaneously increasing the risk of antimicrobial-resistant pathogen carriage, expansion within the microbiome, transmission, and infection [3, 11]. As such, optimization of antibiotic treatment to minimize gut microbiome disruption and ARG carriage may protect against antimicrobial resistance. The BALANCE randomized controlled trial (RCT) recently compared 7 and 14 days of antibiotic treatment for bacteremia in the largest study of its kind to date and found that 7 days of treatment was noninferior for the outcome of 90-day survival [12]. However, it is not known whether shorter durations of therapy decrease harm to the microbiome or decrease selection for ARGs within it. In this substudy, we evaluated the impact of antibiotic duration on the gut microbiome and ARG carriage in a subset of participants in the BALANCE RCT. We hypothesized that antibiotic-associated gut microbiome disruption would be less pronounced in participants who received 7 days of antibiotic as compared with 14 days.

## METHODS

### Study Design

The multinational BALANCE clinical trial enrolled 3631 participants with bacteremia across 74 hospitals and randomized them 1:1 to receive 7 or 14 days of antibiotics to assess noninferiority of shortened antibiotic duration. Participants were eligible for enrollment into the BALANCE trial if they were admitted to hospital and had a positive blood culture result with a pathogenic bacterial species other than common contaminant organisms (eg, coagulase-negative staphylococci, *Bacilli* spp, *Corynebacterium* spp, *Propionobacterium* spp, *Aerococcus* spp, or *Micrococcus* spp), *Staphylococcus aureus*, *Staphylococcus lugdunensis*, *Candida* spp, or other fungal species. While the duration of treatment was determined by randomization, selection of antibiotic regimens, including antimicrobial agents, doses, frequency, and route of delivery, was at the discretion of the treating physicians. Complete trial details can be found in the BALANCE trial and protocol publications [12, 13]. For this substudy, rectal swabs were collected from a subset of 131 trial participants from 5 Ontario-based study sites who agreed to participate in this optional substudy. Swabs were collected on the day of enrollment (median antibiotic treatment day, 3; IQR, 2–5) and antibiotic treatment days 7, 14, and 21 or discharge from hospital, whichever was earlier. Immediately following collection, swabs were immersed in DNA/RNA Shield and then frozen or frozen dry. All swabs were frozen within 2 hours of collection at −20 or −80 °C depending on freezer availability at the study site.

### Patient Consent Statement

Informed consent for inclusion in this substudy was obtained for all participants at each of the 5 study sites: Sunnybrook Health Sciences Centre, St Michael's Hospital, Mount Sinai Hospital, Queen's University, and Niagara Health.

### DNA Extraction and Metagenomic Sequencing

DNA was extracted from rectal swabs with the DNeasy PowerSoil Pro Kit (Qiagen) according to the manufacturer's instructions. Prior to metagenomic sequencing, libraries were prepared with Illumina DNA Prep kits. Paired-end metagenomic sequencing with a 150–base pair insert size was performed with the Illumina NovaSeq X platform. Nextera adapters were removed from reads with Trimmomatic version 0.39. Human and phiX reads were removed by Kneaddata version 0.7.2. Taxonomic characterization of reads was performed with Metaphlan version 4.0.6 with default parameters [14]. To filter out low-quality samples, only samples with >100 000 metagenomic reads were included in downstream analyses. All taxonomic profiles were collapsed to the species level for downstream analyses. For all alpha diversity analyses (eg, Shannon diversity, species richness, evenness, and Berger-Parker index), samples were subsampled to 100 000 reads with the “rarefy” function in the *vegan* package in R. To characterize ARGs, metagenomic reads were mapped to CARD with RGI bwt version 6.0.1 with default parameters [15]. To account for differences in ARG length and sequencing depth, ARG abundance was expressed as reads per kilobase per million.

### Outcomes

The primary outcome for this subanalysis was change in gut microbiome Shannon diversity, a composite ecologic index of microbial richness and evenness, at day 7 vs day 14. Shannon diversity is a composite index incorporating the following: *taxonomic richness*, the number of unique taxa detected at least once in the community, and *evenness*, an indicator of uniformity of taxonomic relative abundances, with higher evenness indicating more uniform relative abundance distribution. Shannon diversity was chosen as the primary outcome as it is a widely used global indicator of gut microbiome composition. Secondary outcomes included assessments of other dimensions of gut microbiome composition and correlates of antimicrobial resistance in the gut microbiome from day 7 to 14: first, the within-sample/alpha diversity indicators of species richness (the number of unique species), evenness [16], and Berger-Parker dominance (the relative abundance of the most abundant taxon, with higher values indicative of a highly dominated/disrupted microbiome) [17]; second, ARG abundance (defined as total reads per kilobase per million of all ARGs). Additional secondary outcomes included gut microbiome alpha diversity metrics and ARG abundance at day 14. Post hoc analyses were also performed to assess whether there was a significant change in metagenomic features from enrollment to day 7.

### Statistical Analysis

Comparisons of continuous variables were performed with the Mann-Whitney *U* test and categorical variables with Pearson χ^2^ test. The Wilcoxon signed rank test was used to compare continuous variables against a hypothetical value of 0. Linear regression was used to assess the association between study group and metagenomic features while controlling for additional covariates. Differential abundance analysis for bacterial species and ARG was performed with MaAsLin2 version 1.16.0 with treatment group as the fixed effect and, if multiple time points per patient were included, patient identification as the random effect [18]. A Bray-Curtis dissimilarity matrix was generated with the *vegan* package (version 2.6-6.1) in R [19] and used as the input for principal coordinate analysis with the *ecodist* package (version 2.1.3) in R [20]. Permutational multivariate analysis of variance that accompanies principal coordinate analysis was performed with the *vegan* package in R to compare beta diversity between groups/samples. To compare microbiome dissimilarity from day 7 to 14 between treatment groups, Bray-Curtis dissimilarities between days 7 and 14 for each participant were extracted from the total dissimilarity matrix. Based on an expected difference in Shannon diversity of 10% between groups and an SD of 40%, our sample size calculation indicated a required sample size of 125 participants with a 2-sided α of .05 and β of 80%. A 10% difference in Shannon diversity was chosen as a conservative threshold to ensure an adequate sample size, since previous reports have shown >10% loss of Shannon diversity following antibiotic treatment [21]. All statistical tests and graphical presentation of results were performed with RStudio (R version 4.3.2) or Prism version 10.2.0 (GraphPad). All accompanying R code is available from the corresponding author upon request.

## RESULTS

### Participant Characteristics

**Figure 1.**
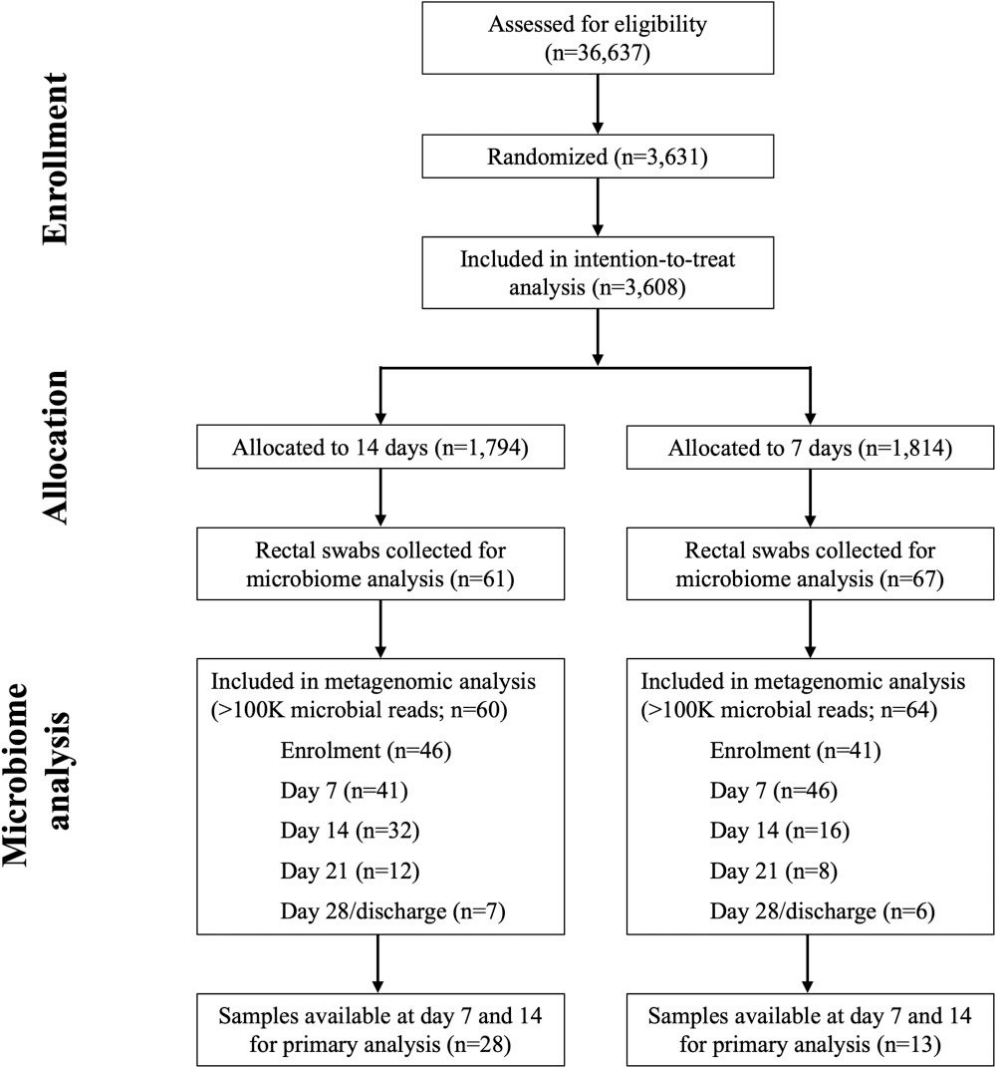
CONSORT diagram of BALANCE randomized controlled trial enrollment and inclusion in the microbiome substudy. BALANCE ClinicalTrials.gov NCT03005145 [12].

Among the 3631 participants with bacteremia in the BALANCE RCT who were randomized (Figure 1), rectal swab samples were collected from a subset of 131 participants for characterization of gut microbiome taxonomic composition and ARG carriage by metagenomic sequencing. We retained metagenomic data from 124 participants after quality control processing of metagenomic samples: 60 (48%) received 14 days of antibiotics and 64 (52%) received 7 days. Forty-one participants (n = 28 in the 14-day group, n = 13 in the 7-day group) had samples available from days 7 and 14 for our primary comparison. Baseline characteristics for all participants in this metagenomic substudy and those in the primary analysis did not significantly differ between groups. Piperacillin-tazobactam exposure up to day 14 was higher in the 7-day group for the metagenomic study population (69% vs 51%, *P* = .05) and the participants in the primary analysis (69% vs 46%), although the latter did not reach statistical significance (*P* = .30; Table 1).

**Table 1.** Baseline BALANCE Randomized Controlled Trial Participant Characteristics

	All Participants in Metagenomic Substudy			Participants in Primary Analysis (Paired Day 7 and 14 Samples)		
	14-d Group (n = 60)	7-d Group (n = 64)	*P* value	14-d Group (n = 28)	7-d Group (n = 13)	*P* Value
Age, y	66.5 (60.5–82.25)	71.5 (60–79.25)	.26	65 (57–71.25)	62 (56–71)	.58
Birth sex			>.99			.86
Male	34 (57)	36 (56)		17 (61)	9 (69)	
Female	26 (43)	28 (44)		11 (39)	4 (31)	
Baseline mechanical ventilation	30 (50)	24 (38)	.22	19 (68)	10 (77)	.82
Comorbidity						
Diabetes mellitus	12 (20)	15 (23)	.92	8 (29)	5 (38)	.79
Solid malignancy	12 (20)	21 (33)	.21	5 (18)	3 (23)	>.99
Obesity	6 (10)	7 (11)	>.99	2 (7)	1 (8)	>.99
Arrhythmia	4 (7)	9 (14)	.34	1 (4)	1 (8)	>.99
Corticosteroid or other immunosuppressant	4 (7)	6 (9)	.89	1 (4)	0 (0)	>.99
Chronic obstructive pulmonary disease	4 (7)	3 (5)	.87	1 (4)	1 (8)	>.99
Renal insufficiency	5 (8)	4 (6)	.86	5 (18)	0 (0)	.27
Congestive heart failure	4 (7)	10 (16)	.23	3 (11)	1 (8)	>.99
Liver disease	3 (5)	5 (8)	.84	2 (7)	1 (8)	>.99
Peripheral vascular disease	2 (3)	3 (5)	>.99	2 (7)	0 (0)	.83
Dialysis dependency	0 (0)	4 (6)	.16	0 (0)	1 (8)	.69
Leukemia or lymphoma	3 (5)	3 (5)	>.99	2 (7)	0 (0)	.83
Any source control procedure	41 (68)	32 (50)	.06	22 (79)	7 (54)	.21
Antimicrobial exposure up to day 14						
Piperacillin-tazobactam	31 (52)	45 (70)	.05	13 (46)	9 (69)	.30
Ceftriaxone	28 (47)	29 (45)	>.99	14 (46)	5 (38)	.89
Vancomycin	24 (40)	23 (36)	.78	12 (43)	8 (62)	.44
Meropenem	24 (40)	20 (31)	.41	16 (57)	7 (54)	>.99
Ciprofloxacin	20 (33)	19 (30)	.81	7 (25)	3 (23)	>.99
Cefazolin	11 (18)	15 (23)	.63	6 (21)	6 (46)	.21
Other	51 (85)	47 (73)	.17	22 (79)	9 (69)	.80

Data are presented as median (IQR) or No. (%). *P* values obtained with the Mann-Whitney *U* test for continuous variables or the χ^2^ test for categorical variables. BALANCE ClinicalTrials.Gov: NCT03005145 [12].

### Impact of Antibiotic Duration on Gut Microbiome Disruption

**Figure 2.**
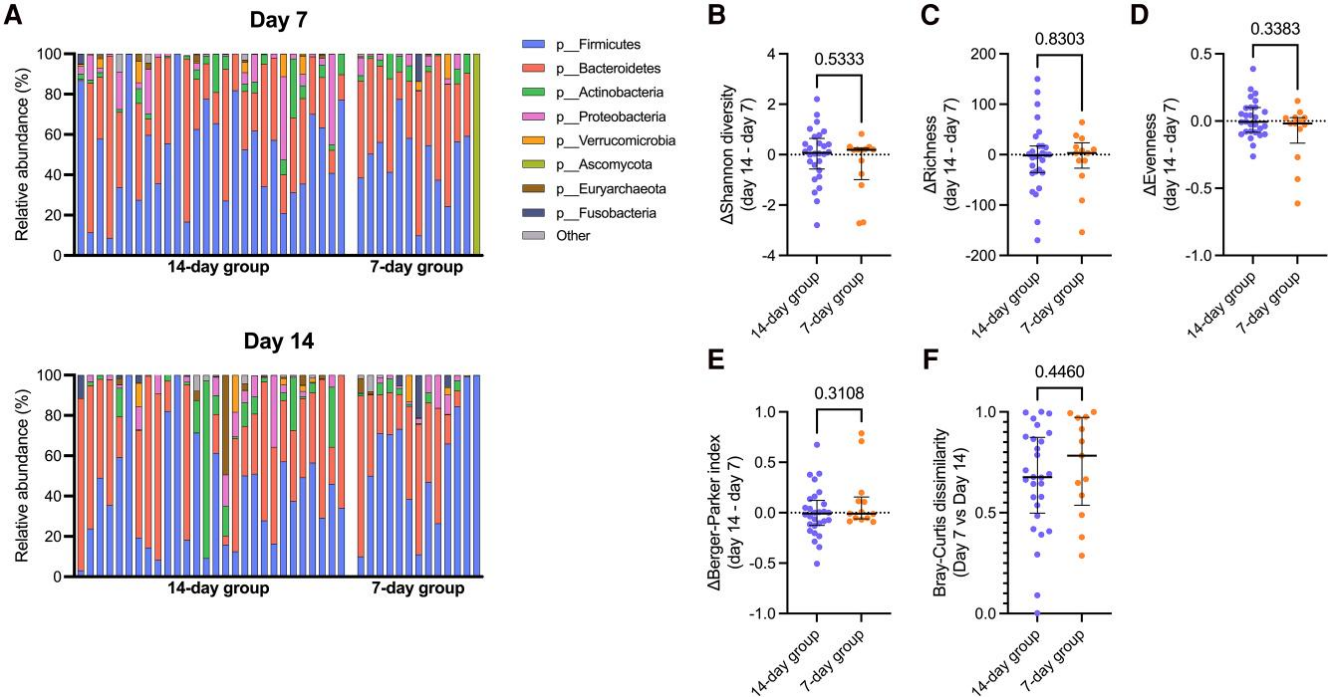
Impact of antibiotic treatment duration on gut microbiome composition. *A*, Gut microbiome composition at the phylum level in both treatment groups at days 7 and 14. Comparison of change in gut microbiome from day 7 to 14 between treatment groups: *B*, Shannon diversity; *C*, species richness; *D*, evenness; and *E*, Berger-Parker index. *F*, Comparison of Bray-Curtis dissimilarity of day 7 and 14 samples between treatment groups. *B–F*, Data are presented as median (IQR). *P* values obtained with the Mann-Whitney *U* test.

**Figure 3.**
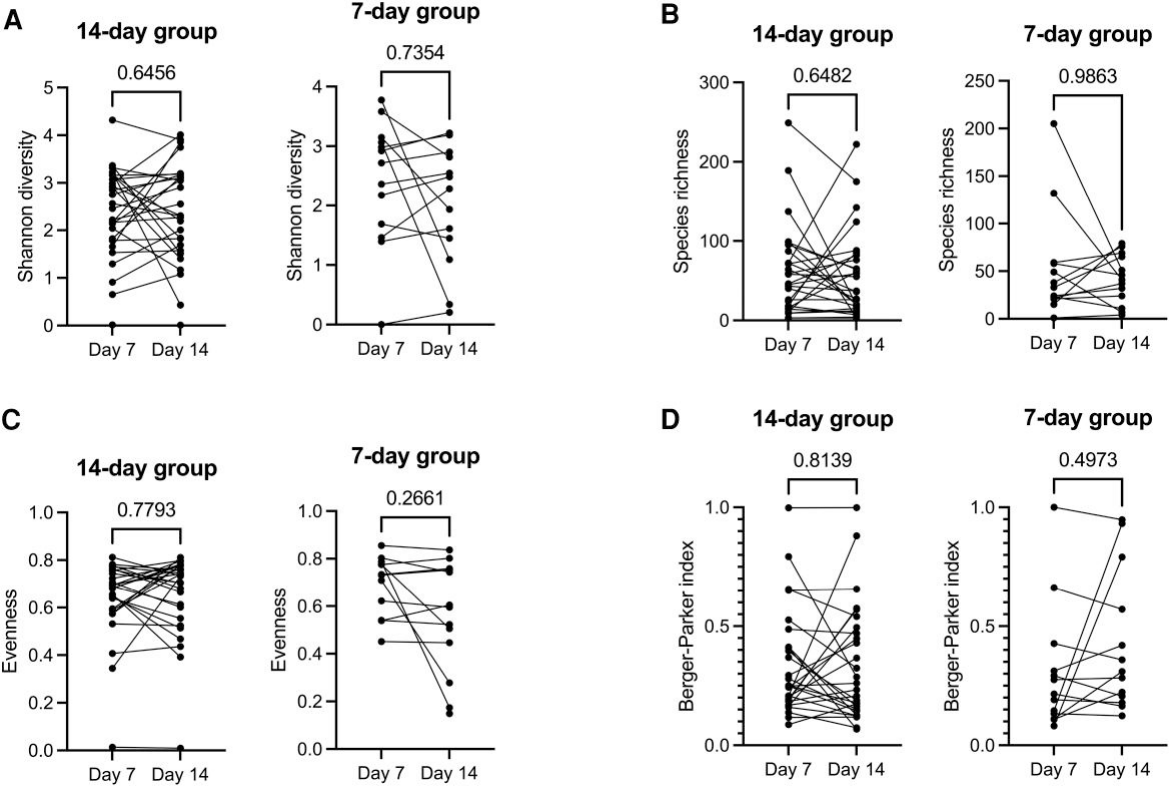
Change in gut microbiota composition from day 7 to 14 based on antibiotic duration. Comparison of gut microbiota between days 7 and 14 in both treatment groups: *A*, Shannon diversity; *B*, species richness; *C*, evenness; and *D*, Berger-Parker index. *P* values obtained with the Wilcoxon matched pairs signed-rank test.

To control for differences in the gut microbiome between groups prior to divergence of treatment regimen (ie, continuation vs discontinuation of treatment at day 7), we calculated the change in gut microbiome taxonomic diversity between days 7 and 14. Antibiotic duration had no significant effect on change in Shannon diversity (*P* = .53), species richness (*P* = .83), evenness (*P* = .34), or Berger-Parker dominance (*P* = .31; Figure 2). Gut microbiome taxonomic (Bray-Curtis) dissimilarity between days 7 and 14 did not differ between groups (*P* = .45). From day 7 to day 14, we did not observe any change in gut microbiome diversity metrics in either treatment group (Figure 3). Similar results were observed after controlling piperacillin-tazobactam exposure prior to day 14 (Supplementary Table 1). To assess whether treatment duration had any effect on change in the relative abundance of individual microbes, particularly gut pathogens, we conducted a differential abundance analysis with MaAsLin2 that included measurements from days 7 and 14, with participant as a random effect and study group as a fixed effect. Although several taxa tended to differ between groups, none of these comparisons remained significant after controlling for multiple comparisons (Supplementary Figure 1).

We compared gut microbiome composition at day 14 between groups among all participants with an available sample at day 14. We observed a trend of lower gut microbiome evenness in the 7-day group (*P* = .06), although overall microbiome composition did not differ between groups (permutational multivariate analysis of variance: *R*^2^ = 0.02, *P* = .87). Relative abundance of individual microbial species did not differ between groups after correcting for multiple comparisons (Supplementary Figure 2). Due to a greater proportion of patients in the 7-day group being discharged from the hospital prior to the visit at day 14 (41/65, 63%) as compared with the 14-day group (22/61, 36%, *P* = .004; Supplementary Table 2), fewer samples were collected in the 7-day treatment group at day 14 (n = 16) vs the 14-day treatment group (n = 32).

### Gut ARG Carriage and Antibiotic Duration

**Figure 4.**
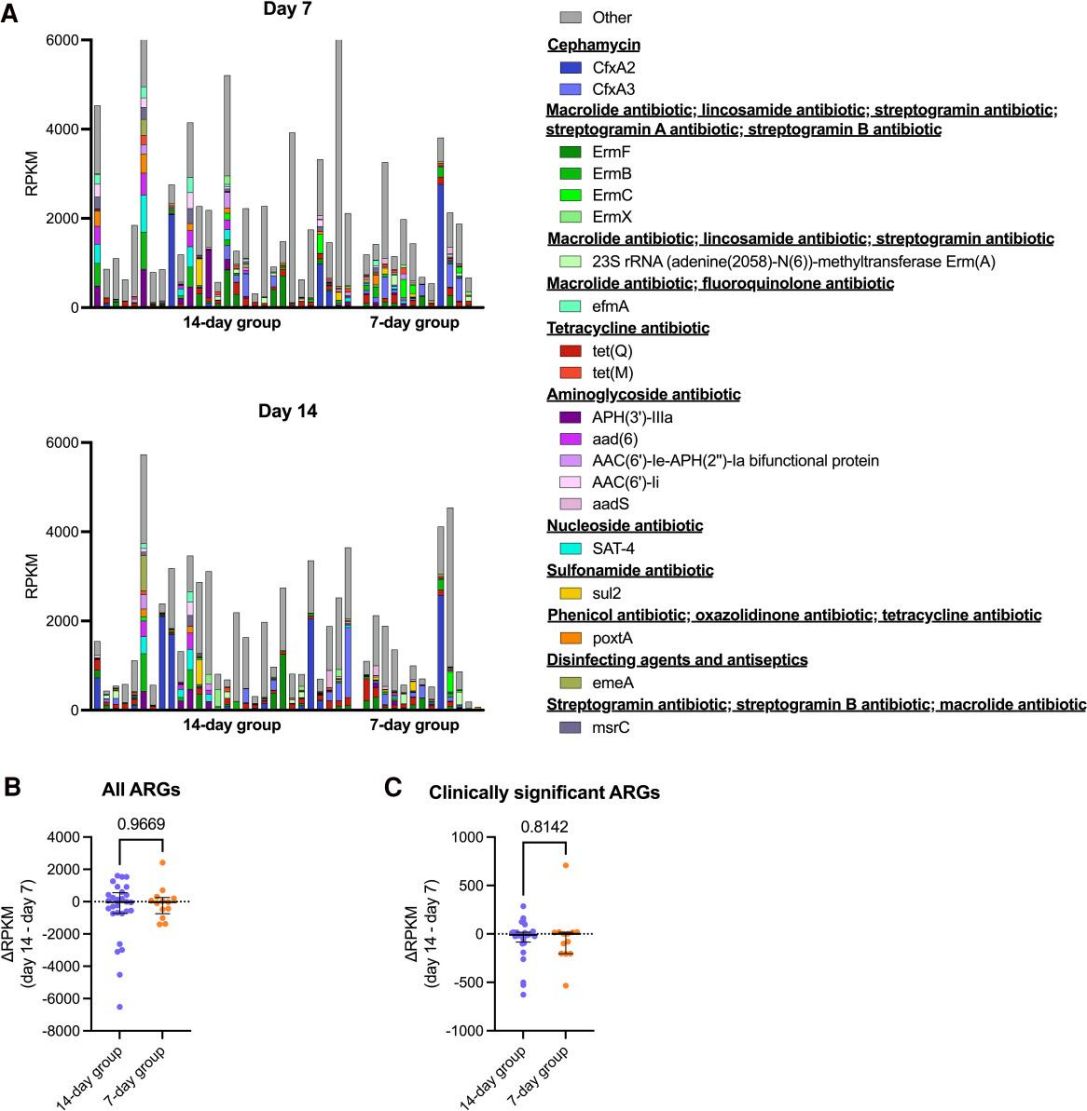
Impact of antibiotic treatment duration on gut resistome composition and ARG burden. *A*, Overall composition of the gut resistome at the ARG level at days 7 and 14 in both treatment groups. Change from day 7 to 14 between treatment groups: *B*, total ARG abundance; *C*, clinically significant ARG abundance. *B–C*, Data are presented as median (IQR) *P* values obtained with the Mann-Whitney *U* test. ARG, antimicrobial resistance gene; RPKM, reads per kilobase per million.

Change in the overall burden of ARGs from day 7 to day 14 did not differ between groups, nor did the abundance of individual ARGs after correcting for multiple comparisons (Figure 4, Supplementary Figure 3). There was no change in total ARG abundance from day 7 to day 14 in either treatment group (Supplementary Figure 4). We also selected a subset of ARGs based on their clinical significance (Supplementary Table 3) for further analysis, which included ARGs that confer resistance to aminoglycosides, cephalosporins, β-lactam/β-lactamase inhibitor combinations, methicillin (for *S aureus*), carbapenems, fluoroquinolones, tetracyclines, macrolides, and vancomycin. Change in abundance of clinically significant ARGs from day 7 to 14 did not differ between groups. Neither treatment group exhibited a change in total clinically significant ARG abundance from day 7 to 14 (Figure 2, Supplementary Figure 4). We did not observe any difference in the prevalence of each clinically significant ARG from day 7 to 14 in either group (Supplementary Figure 5).

### Gut Microbiome Composition and ARG Carriage at Enrollment vs Day 7

Given our observation that an additional 7 days of antibiotic treatment did not result in changes in the gut microbiome (composition or ARG content) in the group receiving 14 days of antibiotics, we hypothesized that antibiotic-induced gut microbiome disruption occurs prior to day 7 and then stabilizes. Baseline samples, which were collected within the first 7 days of antibiotic treatment, were analyzed to assess the short-term impact of antibiotic treatment on the gut microbiome and resistome. Fifty-five participants had samples available from enrollment and day 7. Enrollment samples were collected a median 4 days prior to the day 7 sample (range, 1–7). Gut microbiome Shannon diversity (*P* = .45), species richness (*P* = .13), evenness (*P* = .73), and Berger-Parker index (*P* = .81) did not change between enrollment and day 7. Total ARG abundance (*P* = .19) and abundance of clinically relevant ARGs (*P* = .41) also did not change from enrollment to day 7 (Supplementary Figure 6).

## DISCUSSION

In this metagenomic substudy of the BALANCE RCT, we did not detect greater gut microbiome disruption or ARG carriage after 14 days of antibiotics as compared with 7 days. Although there was a trend of lower gut microbiome disruption in the 14-day group at the end of treatment, this may be confounded by higher rates of hospital discharge prior to this visit in the 7-day group or imbalances in antibiotic exposures (ie, greater exposure to piperacillin-tazobactam in the 7-day group). In post hoc analyses, we did not observe any disruption to the gut microbiome or accumulation of ARGs between enrollment and day 7, suggesting that antibiotic-induced gut microbiome disruption may occur immediately following antibiotic initiation or has already occurred prior to antibiotic initiation. Due to increased discharges in the 7-day treatment group and a small number of samples collected after antibiotic discontinuation, we cannot assess whether there are changes (eg, recovery of microbiome diversity and/or decreases in ARG abundance) that occurred more often or rapidly in patients treated with shorter courses of therapy. That is, it remains possible that 7 days of treatment is associated with more rapid recovery of the microbiome than 14 days of treatment, albeit beyond the time frame that we were able to assess.

Our primary findings that gut microbiome disruption and ARG carriage did not differ by antibiotic duration are consistent with previously published findings. In a prospective cohort study nested within an RCT, Leo et al similarly found that gut microbiome composition and ARG abundance were similar between participants receiving 7 and 14 days of antibiotics for gram-negative bacteremia [22]. In a prospective cohort study evaluating long (>7 days) and short (≤7 days) duration of ciprofloxacin for urinary tract infection, Rodriguez-Ruiz et al found no difference in posttreatment gut microbiome diversity between treatment groups, although longer treatment was associated with significantly reduced gut microbiome diversity when compared with antibiotic-unexposed controls. The authors also observed a sustained increase in ARG abundance in the gut microbiome in the longer treatment group, albeit nonsignificantly [23]. We expand on this by showing that subsequent days of antibiotic treatment after the first week of therapy did not have any effect on gut microbiome diversity, suggesting that antibiotic-induced disruption to the gut microbiome may not be exacerbated by additional days of antibiotic therapy. This is consistent with previous studies that have shown the rapid effects of antibiotics on the gut microbiome of healthy participants, occurring within days of exposure or even after single antibiotic doses [24, 25].

Our analysis has important limitations. First, the sample size for our primary analysis was lower than our target size of 125 participants, despite a total sample of 131 participants, primarily due to the absence of samples at days 7 and 14 for all participants. This may have limited our ability to detect differences in gut microbiome disruption or ARG carriage between treatment groups, particularly if these differences were small in magnitude. Future studies are encouraged to explore these relationships in larger studies from demographically, geographically, and clinically diverse cohorts, since this analysis included only participants from 5 Ontario-based sites. Second, higher rates of piperacillin-tazobactam exposure in the 7-day group may confound duration-specific effects. Piperacillin-tazobactam has been shown to be particularly damaging to the gut microbiome as compared with other antibiotics [26]. If these effects are longer-lasting than other antibiotics, the higher rates of piperacillin-tazobactam in the 7-day group may limit our ability to detect any protective effects of shorter antibiotic duration on the gut microbiome. Third, we had fewer samples in the 7-day treatment group than the 14-day treatment group at day 14 due to a greater number of discharges in this group. Since discharged participants may have been healthier, this may explain the significantly lower microbiome diversity observed in the 7-day group at day 14 (due to enrichment for sicker patients in this group who remained in hospital at this time point) and may miss early microbiome “recovery” after hospital discharge. However, this limitation would not affect our observation that an additional 7 days of antibiotics in the 14-day treatment group was not associated with intensified disruption of microbiomes or additional ARG accumulation. While this potential source of bias may affect the interpretation of our comparisons at day 14, our primary comparison (ie, change in gut microbiome diversity and ARG carriage from day 7 to 14) should minimize the impact of this bias by normalizing to day 7.

Seven days of antibiotics has now been shown to be noninferior to 14 days of therapy for treatment of bacteremia. Here, we did not detect greater disruption to the gut microbiome or accumulation of ARGs after 14 days of antibiotics vs 7 days, potentially because the greatest impact of antibiotics on the gut microbiome and resistome occurs within the first few days of antibiotics.

The limitations of this analysis highlight several important considerations for future studies. To comprehensively characterize the impact of antibiotic treatment on the gut microbiome, our results suggest that sampling prior to antibiotic initiation and within the first few days of antibiotics may be necessary. Understandably, it is difficult to predict the onset of bacteremia and therefore difficult to design a study that includes sampling before and immediately after initiation of antibiotics, thereby emphasizing the need to explore alternative clinical cohorts where the initiation of antibiotics is either more predictable (ie, preplanned) or of sufficient frequency that a cohort study would capture initiation events. Studies would also be optimally designed to include postdischarge samples to assess microbiome recovery after hospital discharge, which may feature the incorporation of at-home sample collection strategies.

## Supplementary Material

ofaf137_Supplementary_Data

## Supplementary Data

Supplementary materials are available at *Open Forum Infectious Diseases* online. Consisting of data provided by the authors to benefit the reader, the posted materials are not copyedited and are the sole responsibility of the authors, so questions or comments should be addressed to the corresponding author.

## Note

***Financial support*.** This work was supported by the Canadian Institutes of Health Research and Physicians’ Services Incorporated Foundation (grants PJT-365513 and PJT-176342); and the Toronto General Hospital Research Institute to E. A. (Postdoctoral Researcher Award).
