# Supplementary material for: Impact of Antibiotic Duration on Gut Microbiome Composition and Antimicrobial Resistance: A Substudy of the BALANCE Randomized Controlled Trial
Source: Open Forum Infect Dis. 2025 Mar 14;12(3):ofaf137. doi: 10.1093/ofid/ofaf137 (PMC11935739; doi:10.1093/ofid/ofaf137)
Supplement: ofaf137_Supplementary_Data [file ofaf137_supplementary_data.docx]

**SUPPLEMENTAL FIGURES**


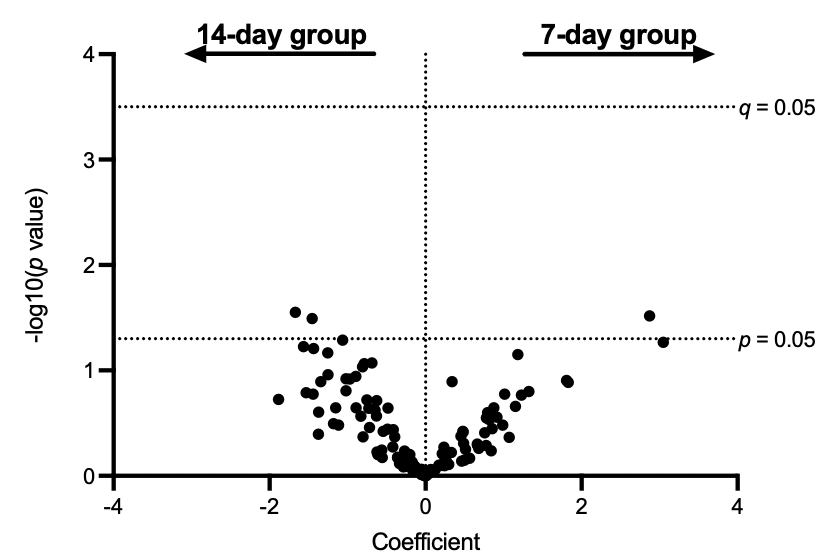


**Figure S1: Differentially abundant microbial species between treatment groups across days 7 and 14.** Volcano plot displaying test coefficients and log-transformed *p*-values for MaAsLin2 analysis that assessed the relationship between microbial species and treatment group, including data from day 7 and day 14 and patient ID as random effect. Positive coefficients represent a positive association with the 7-day group. *P* values adjusted for multiple comparisons with the false discovery rate.

**Figure S1 alt text:** Graph displaying the direction and significance of associations between the relative abundance of individual gut microbes and treatment group.


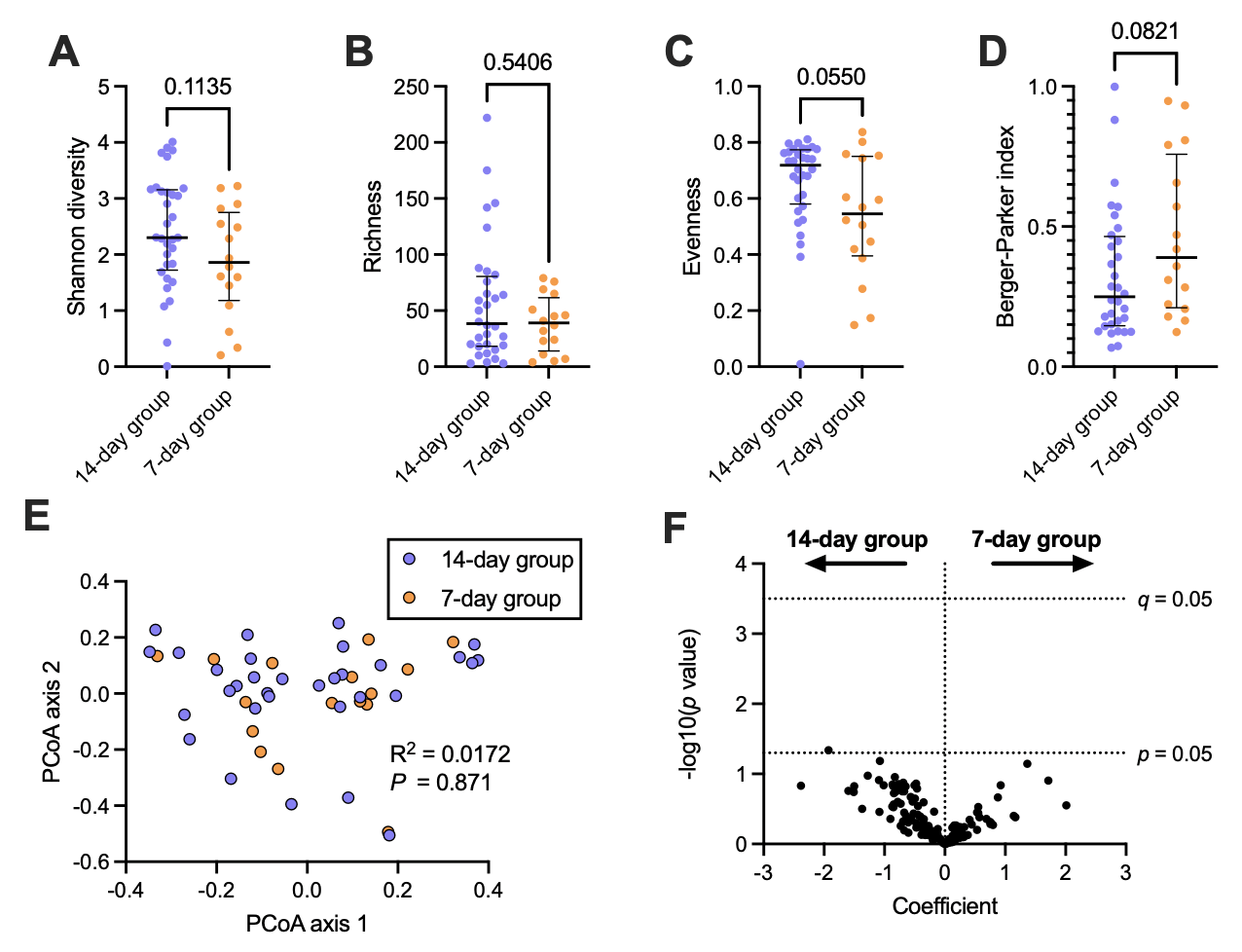


**Figure S2: Cross-sectional comparison of gut microbiota composition between treatment groups at day 14.** Comparison of gut microbiota A) Shannon diversity, B) species richness, C) evenness, and D) Berger-Parker index at day 14 between treatment groups. E) PCoA plot of species-level gut microbiota composition in each treatment group at day 14. F) MaAsLin2 analysis of differentially abundant microbial species at day 14 between treatment groups. *P* values and test statistics obtained with the Mann-Whitney U test, ANOSIM, and MaAsLin2. *P* values from MaAsLin2 analysis adjusted for multiple comparisons with the false discovery rate.

**Figure S2 alt text:** Graphs comparing gut microbiome composition between treatment groups at day 14.


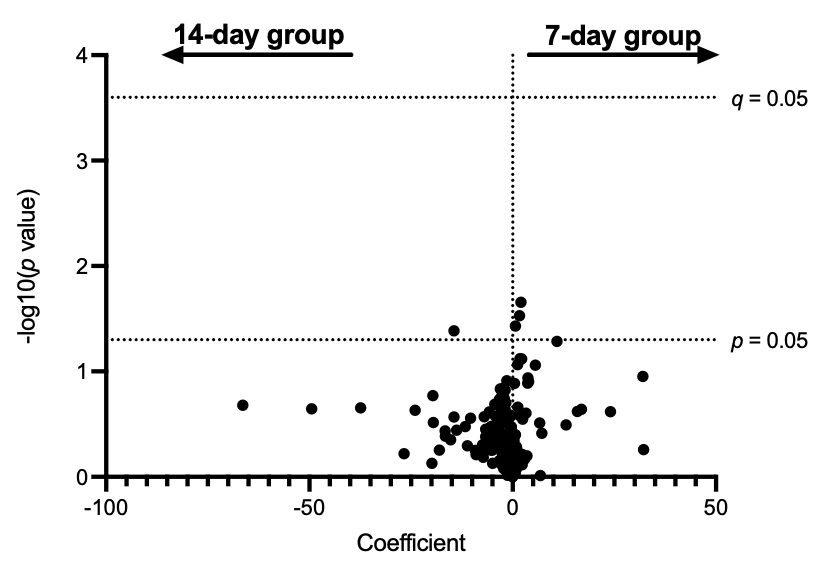


**Figure S3: Differentially abundant ARGs between treatment groups across days 7 and 14.** Volcano plot displaying test coefficients and log-transformed *p*-values for MaAsLin2 analysis that assessed the relationship between ARGs and treatment group, including data from day 7 and day 14 and patient ID as random effect. Positive coefficients represent a positive association with the 7-day group. *P* values adjusted for multiple comparisons with the false discovery rate.

**Figure S3 alt text:** Graph displaying the direction and significance of associations between antimicrobial resistance gene abundances and treatment group.

**
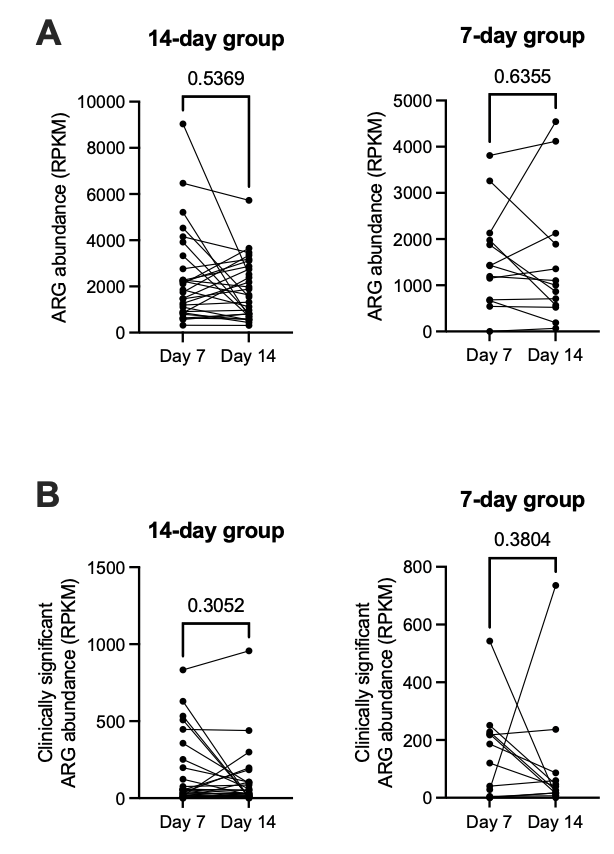
**

**Figure S4: Impact of antibiotic duration on gut resistome dynamics from day 7 to 14.** Comparison of A) total ARG abundance and B) clinically significant ARG abundance between day 7 and 14 in each treatment group. *P* values obtained with the Wilcoxon matched pairs signed-rank test.

**Figure S4 alt text:** Graph displaying a comparison of antimicrobial resistance gene abundance between day 7 and 14 in both treatment groups.


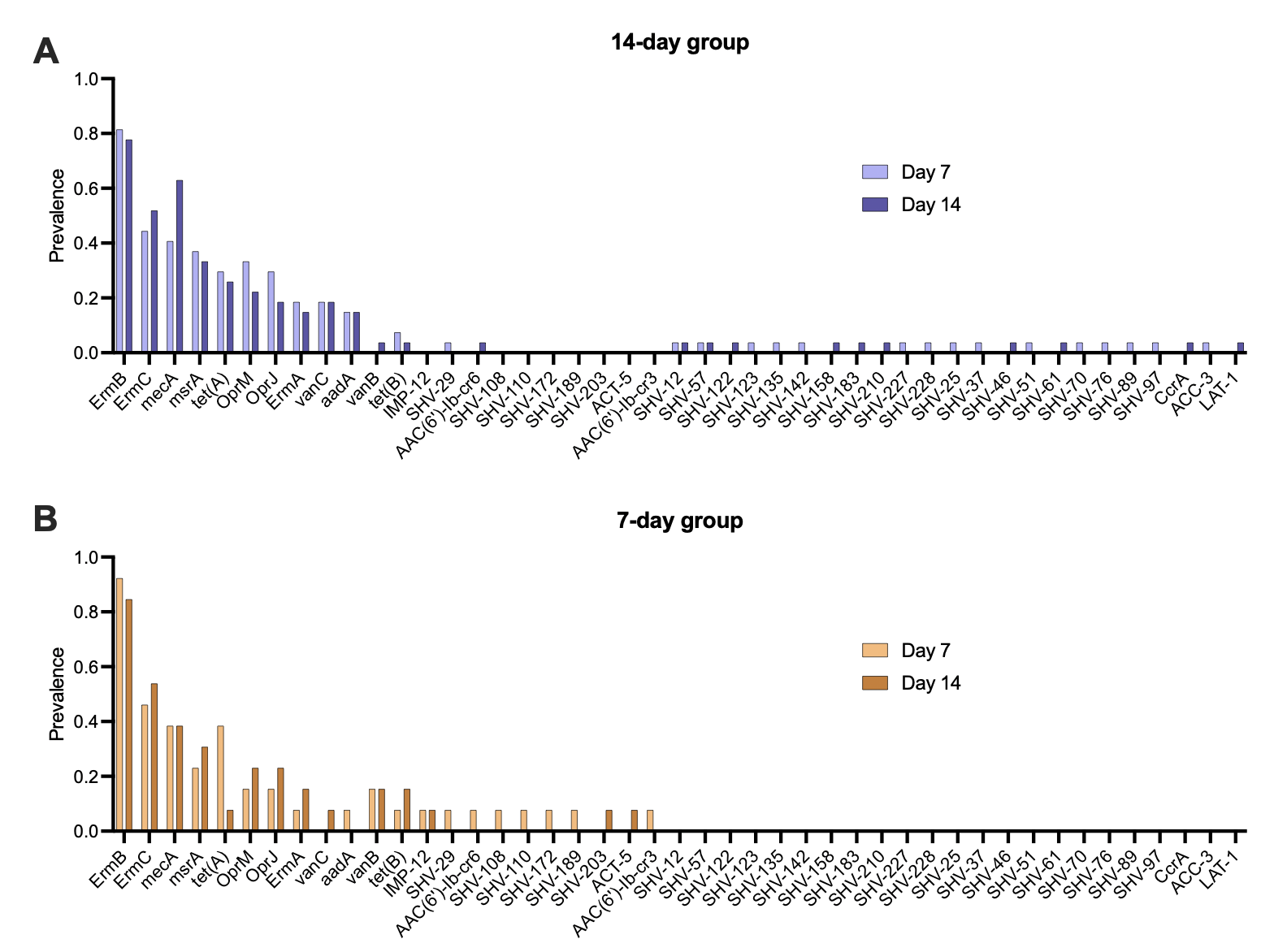


**Figure S5: Impact of antibiotic duration on prevalence of clinically significant ARGs.** Paired comparison of clinically significant ARG prevalences between day 7 and 14 in the A) 14-day group and B) 7-day group. *P* values obtained with the McNemar test. Non-significant *p* values not included on figure.

**Figure S5 alt text:** Graphical representation of antimicrobial resistance gene abundances at day 7 and 14 in both treatment groups.


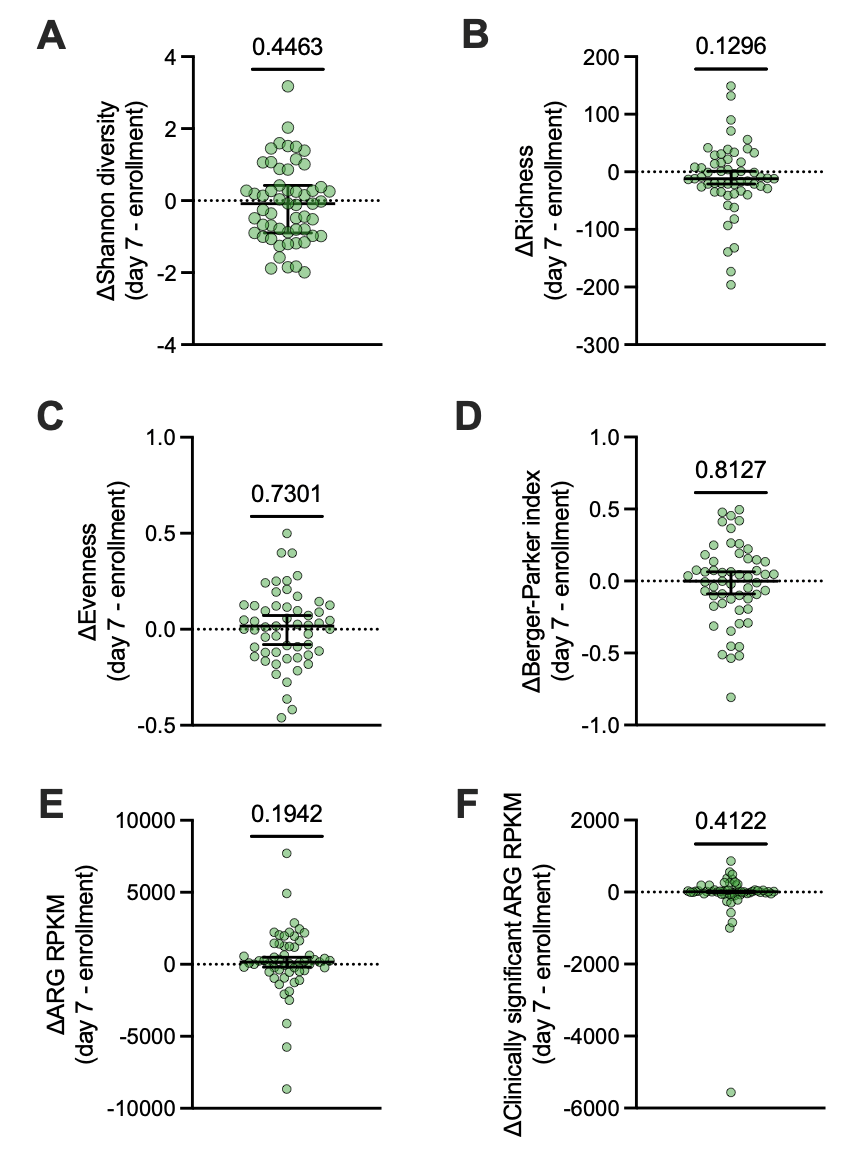


**Figure S6: Change in gut microbiome diversity and ARG carriage during first week of antibiotics.** Difference in gut microbiome A) Shannon diversity, B) species richness, C) evenness, D) Berger-Parker index, E) ARG abundance, and F) clinically significant ARG abundance between day 7 and enrollment visit. *P* values obtained with the Wilcoxon signed rank test comparing to a hypothetical value of 0.

**Figure S6 alt text:** Graphs displaying the change in gut microbiome composition and antimicrobial resistance gene abundance from enrolment to day 7.

**SUPPLEMENTAL TABLES**

**Table S1: Association between treatment group and change in metagenomic features between day 7 and 14 while controlling for piperacillin-tazobactam exposure prior to day 14.** *P* values and test statistics obtained with linear regression.

|  | **Dependent variables** | | | | | | | | | |
| --- | --- | --- | --- | --- | --- | --- | --- | --- | --- | --- |
|  | **ΔShannon diversity**  **(day 7 vs day 14)** | | **ΔSpecies richness**  **(day 7 vs day 14)** | | **ΔEvenness**  **(day 7 vs day 14)** | | **ΔBerger-Parker index**  **(day 7 vs day 14)** | | **Bray-Curtis dissimilarity**  **(day 7 vs day 14)** | |
| **Independent variables** | **Estimate** | ***P* value** | **Estimate** | ***P* value** | **Estimate** | ***P* value** | **Estimate** | ***P* value** | **Estimate** | ***P* value** |
| **Study group**  **(reference: 14-day group)** | 0.36 | 0.35 | 4.67 | 0.84 | 0.10 | 0.09 | -0.10 | 0.25 | -0.05 | 0.60 |
| **Piperacillin-tazobactam exposure prior to day 14** | -0.16 | 0.66 | -7.27 | 0.73 | -0.01 | 0.86 | 0.05 | 0.56 | 0.13 | 0.13 |

**Table S2: Comparison of hospital discharge rates prior to day 14 in each treatment group.** *P* value obtained with the chi-squared test.

|  | **14-day group** | **7-day group** | ***P* value** |
| --- | --- | --- | --- |
| **Discharged before day 14** | 21 (35%) | 40 (63%) | 0.004 |
| **Discharged after day 14** | 39 (65%) | 24 (37%) |  |

**Table S3: Full list of clinically significant ARGs.**

| **Antibiotic class** | **Antibiotic resistance genes** |
| --- | --- |
| Aminoglycosides | aphA6 |
|  | aadA1 |
|  | aacC4 |
|  | aacC2 |
|  | aacC1 |
| Beta-lactams | mecA |
|  | BES-1 |
|  | CTX-M1/8/9 |
|  | GES |
|  | IMI/NMC-A |
|  | KPC |
|  | SHVs |
|  | ccrA |
|  | IMP-1/2/5/12 |
|  | NDM |
|  | VIM-1/7/13 |
|  | ACC-1/3 |
|  | ACT-1/5/7 |
|  | CFE-1 |
|  | CMY-10 |
|  | DHA |
|  | FOX |
|  | LAT |
|  | MIR |
|  | OXA-2/10/18/23/24/45/48/50/51/54/55/58/60/60/62 |
| Fluoroquinolones | QnrA |
|  | B-4/B-5/B-8/B-31/C/D/S |
|  | QepA |
|  | AAC |
|  | oprM |
|  | oprj |
| Other | msrA |
|  | mefA |
|  | ermC |
|  | ermB |
|  | ermA |
|  | vanB/C |
|  | tetA/B |
